# Elicitor-Induced Metabolomics Analysis of *Halodule pinifolia* Suspension Culture for an Alternative Antifungal Screening Approach against *Candida albicans*

**DOI:** 10.3390/jof8060609

**Published:** 2022-06-07

**Authors:** Mousa A. Alghuthaymi, Jeyapragash Danaraj, Fawziah M. Albarakaty, Rajiv Periakaruppan, Manigandan Vajravelu, Saravanakumar Ayyappan, Kumaralingam Selvaraj, Kamel A. Abd-Elsalam

**Affiliations:** 1 Biology Department, Science and Humanities College, Shaqra University, Alquwayiyah 11726, Saudi Arabia; malghuthaymi@su.edu.sa; 2 Centre for Ocean Research (DST-FIST Sponsored Centre), MoES-Earth Science and Technology Cell (Marine Biotechnological Studies), Col. Dr. Jeppiaar Research Park, Sathyabama Institute of Science and Technology, Chennai 600119, India; marinekumar@gmail.com; 3 Department of Biology, Faculty of Applied Science, Umm Al-Qura University, Makkah Al Mukarramah P.O. Box 715, Saudi Arabia; 4 Department of Biotechnology, Karpagam Academy of Higher Education, Eachanari, Coimbatore 641021, India; rajivsmart15@gmail.com; 5 CSIR-RA, International Research Centre, Sathyabama Institute of Science and Technology, Chennai 600119, India; maniraj2227@gmail.com; 6 Centre of Advanced Study in Marine Biology, Faculty of Marine Sciences, Annamalai University, Parangipettai 608502, India; asarvaan@gmail.com; 7 Plant Pathology Research Institute, Agricultural Research Centre, Giza 12619, Egypt; kamelabdelsalam@gmail.com

**Keywords:** *Halodule pinifolia*, Cymodoceaceae, metabolomics, rosmarinic acid, gene expression

## Abstract

Elicitors are the agents that stimulate the defense responses of plants, and accumulate specialized metabolites in plant tissue culture. This study investigated the elicitor-feeding response of *H. pinifolia* suspension cell cultures (SCC) for metabolomics analysis and screening of specialized compounds against *Candida albicans*. Methyl jasmonate (MeJA) was used as an elicitor, and treatment of SCC at a concentration of 20 µM MeJA resulted in the maximum rosmarinic acid (RA) accumulation (117 mg/g dry weight), with transcript levels of RA biosynthetic genes HpPAL, HpC4H, and Hp4CL being 4.2, 2.5, and 3.7-fold higher, respectively, than the controls. GC-MS-based metabolomics analysis revealed a total of 47 metabolites, including 30 organic acids, six amino acids, six flavonoids, two sugars, two plant growth regulators, and one vitamin, which were significantly different between control and MeJA-treated cells. Furthermore, five phenolic acids were discovered at higher concentrations, including *p*-anisic acid, *p*-coumaric acid, caffeic acid, vanillic acid, and rosmarinic acid, and were purified and structurally elucidated for alternative antifungal screening against *C. albicans* and the evaluation of ADMET properties. The results from antifungal screening revealed that RA at MIC of 31.25 mg/L exhibited the lowest growth percentage of *C. albicans* (1.99%), with higher inhibition of isocitrate lyase 1 (ICL 1) enzyme (93.1%), followed by *p*-anisic acid (86.2%) and caffeic acid (85.1%), respectively. The drug likeliness and ADMET properties of RA exhibited promising results, with a bioactivity score of 0.57, 0.15, and 0.24 for nuclear receptor ligand, protease inhibitor, and enzyme inhibitor, respectively. Therefore, MeJA appears to have a significant effect on enhanced RA accumulation in *H. pinifoia* cells with phenylpropanoid transcript expression, and acts as an ICL1 inhibitor of *C. albicans*.

## 1. Introduction

Seagrasses are submerged marine angiosperms that have evolved to tolerate marine environmental conditions that are characteristically unsuitable for most angiosperms. Despite seagrass undergoing various stresses, it is fortunate enough to withstand the unfavorable conditions by synthesizing specialized products [1]. Specialized products or metabolites have played a vital role in pharmaceutical sectors since the ancient period. Seagrasses can be considered as a unique ecological group occurring worldwide in different climatic zones, and share their metabolic features with their terrestrial counterparts [2]. The investigation of antimicrobials, antioxidants, metabolite synthesis, and nutrition has brought seagrasses to the spotlight for undescribed bioactive compounds [3]. In recent years, metabolomics tools have been used to detect and quantify the diverse group of metabolites in different cells or tissues of an organism, with high selectivity and sensitivity to determine the metabolite fingerprints [4]. Metabolomics is an unbiased approach which acquires comprehensive information on the low molecular weight metabolite content of the cell, tissue, or organisms, and their composition, which is likely to be altered, owing to different environmental conditions which reflect different genetic backgrounds. In addition, metabolite profiling combined with chemometrics has been proven to be useful in functional genomics research, and allows the classification of samples with diverse biological status, origin, or quality using statistical techniques such as hierarchical cluster analysis and partial least square discriminant analysis (PLS-DA) [5].

Rosmarinic acid (RA) is a specialized plant product commonly found in species of Boraginaceae, and in the subfamily Nepetoideae of Lamiaceae [6]. It was first isolated and characterized by Scarpati and Oriente [7], who named the compound RA, based on its first source of isolation, i.e., *Rosmarinus officinalis*. In monocotyledonous plants, RA has been explored in Zosteraceae [8], Araceae [9], Cannaceae [10], Poaceae [11], Marantaceae [12], Potamogetonaceae [13], and Hydrocharitaceae [14]. It was suggested that RA biosynthesis involves two pathways, the phenylpropanoid pathway and the tyrosine-derived pathway, comprising eight enzymes [15]. As reported by Ellis and Towers [16], RA biosynthesis begins with two aromatic amino acids as precursors, L-phenylalanine and L-tyrosine. Reports on RA have revealed that undifferentiated plant cells accumulate much higher concentrations of RA than wild plants. Moreover, this molecule constituted 98.05% of total phenolics [17]; thus, the production of RA by plant cell suspension culture was first derived in *Coleus blumei* [18,19], and has been intensively studied in suspension-cultured cells of *Salvia officinalis* [20], *Anthoceros agrestis* [21], *Lavandula vera* [22], *Agastache rugosa* [23], and *Ocimum sanctum* [24]. Several factors, such as phytohormones, microelements level, and the presence of precursors from the biosynthetic pathway, affect specialized product accumulation in various in-vitro plant tissue cultures. However, the most important factor in enhancing their synthesis is the elicitation process [25]. The production of many valuable metabolites using various elicitors has been reported [26]. Yeast extract, vanadium sulphate, and methyl jasmonate (MeJA) have been used as elicitors for the production of potential pharmaceutical compounds at higher concentrations. MeJA has been implicated as an intermediate signal for the elicitation of various metabolites, e.g., hypericins and hyperforin [27,28], saponins [29], as well as coumarin derivatives and isoflavonoids [30]. Though the cell suspension culture of *H. pinifolia* was established by Subhashini et al. [31], it has not been further subjected for any other in-vitro experiments, and, to the best of our knowledge, the impact of MeJA on RA production in *H. pinifolia* has not been elucidated.

Despite the increasing demands of plant secondary metabolites, it becomes important to focus on the discovery of plant-based drugs used for the treatment of numerous diseases. Though the antimicrobial, anti-inflammatory, and neuroprotective activities of RA have been documented against Gram-positive and Gram-negative pathogens, there is no clear evidence of RA on the clinical bacterial strains which cause harmful diseases, and, therefore, researchers have turned their attention to understand the bioactive potential of RA for antimicrobial applications. Candidiasis is among the most common nosocomial systemic infections caused by *C. albicans*, with mortality rates as high as 50%. The infection by *C. albicans* has gained increasing importance over the past 2–3 decades, and this trend will continue inexorably into the 21st century. Glycolysis, gluconeogenesis, and the glyoxylate cycle contribute to the survival of *C. albicans* during the infection, but their specific mechanism is poorly understood. Among these metabolic pathways, the glyoxylate cycle has been widespread and well-documented. The key enzymes for this pathway are isocitrate lyase (ICL 1) and malate synthase (MS), reported to be highly conserved among bacteria, plants, fungi, and nematodes. In this pathway, the CO_2_ generation gets bypassed, and the carbon is conserved as substrates for gluconeogenesis, during which, they are incorporated into new molecules of glucose. *C. albicans* conserves carbon by utilizing the alternate carbon sources, such as lipids and amino acids, to survive and grow in a nutrient-deficient environment inside phagocytic cells such as macrophages and neutrophils [32,33]. No human ortholog has been studied for seagrass metabolites using such pathways or their respective enzyme, which makes this a promising antifungal target to treat *C. albicans* infection. Therefore, an alternative antifungal screening approach was executed to identify the new compounds from seagrasses that inhibit antifungal target enzyme isocitrate lyase in *C. albicans*. Since the potential compounds may have highly complex structures with many chiral centers, it has not been attempted for chemical synthesis. So as to conserve the seagrass community and prevent the loss in compound activity during chemical synthesis, the potentially derived compounds or inhibitors from seagrasses need to be biosynthesized by means of plant tissue culture technology. Thus, the study investigated the influence of MeJA on metabolomics variation with enhanced RA biosynthesis using suspension-cultured cells of the tropical seagrass *Halodule pinifolia* (Miki) Hartog of the Cymodoceaceae family for an antifungal screening approach against *C. albicans*, and in-silico analysis.

## 2. Materials and Methods

### 2.1. Plant Material

Young meristematic plants (rhizomes) of *Halodule pinifolia* (Miki) Hartog (Cymodoceaceae) were collected in February 2021 from the Vellar estuary (11°29′30.5″ N; 79°46′30.6″ E), Southeast coast of India. The collected samples were washed with native estuary water to remove epiphytes and sand particles, and then washed several times with autoclaved seawater (salinity of 30 psu). It was transported to the laboratory for callus induction.

### 2.2. Surface Sterilization of Explant

Surface sterilization of the explant was performed following the methodology of Jeyapragash et al. [34] with slight modifications. Rhizomes of *H. pinifolia* were used as explants, which was aseptically excised (5 mm), surface sterilized (ethanol; 70%, *v*/*v*) for 2 min, and washed thrice with sterile seawater. The explant was soaked in antibiotic solution composed of Nystatin (120 mg L^−1^), Gentamycin (60 mg L^−1^), and Kanamycin (60 mg L^−1^) for 24 h with mild agitation and washing with the sterile seawater.

### 2.3. Callus Induction and Suspension-Cultured Cells (SCC) of H. pinifolia

Callus induction from *H. pinifolia* was established according to the method of Subhashini et al. [32] and Jeyapragash et al. [34]. A rhizome-derived callus was grown on MS agar medium supplemented with 4% sucrose, 0.8% agar, and plant growth regulators (1.5 mg/L 2, 4-D, and 1 mg/L 6-BAP). The medium was prepared using autoclaved seawater (30 ppt), whereas the pH of the medium was adjusted to 5.6 with 1 N HCl and autoclaved at 121 °C for 15 min. The cultures were incubated at 23 ± 2 °C with 16 h photoperiod under white fluorescent light of 50 µmol m^−2^ s^−1^ (40 W cool-white fluorescent tubes, Philips Electronics India Ltd.) and 50–60% relative humidity.

The Calli developed after two weeks at the edges of the explants were used for the establishment of suspension-cultured cells. The cellular suspension of *H. pinifolia* was initiated by transferring the friable calli grown in MS agar medium to MS basal liquid medium with the above-mentioned hormone concentration. The culture flasks were incubated with continuous shaking on a rotary shaker at 120 rpm under the same culture conditions. All experiments were carried out in triplicate.

### 2.4. Preparation of MeJA

A stock solution of MeJA (50 µM) was prepared using 96% ethanol and filter sterilized with a syringe filter (20 µm). MeJA, at a concentration of 20 µM, were prepared from the stock solution and added aseptically to the 10-day-old culture. GC-MS based metabolomic analysis was performed using 21st day old grown culture to identify the the stress responsive metabolites of *H. pinifolia* SCC upon MeJA elicitation.

### 2.5. Extraction of H. pinifolia SCC and Derivatization

The solvents used for metabolite extraction were chosen based on the polarity of the solute of interest. Extraction was carried out with six biological replicates using methanol (MeOH), as per the previous reports of Jeyapragash et al. [34], Abdelfattah El Moussaoui et al. [35], and El Ouadi et al. [36]. Suspension-cultured cells (SCC; 10 gm) of *H. pinifolia* stored at −80 °C were macerated using liquid nitrogen and transferred to 10 mL centrifuge tubes. Methanol (50 mL; precooled at −20 °C) was added to each tube and vortexed for 20 s. The tubes were shaking in a thermomixer at 40 °C for 10 min and centrifuged for 10 min at 11,000× *g*. The supernatant was transferred to glass vial (sterile) and concentrated by vacuum evaporation at 40 °C. Derivatization of SCC extracts were performed for 120 min at 37 °C by adding 80 µL of *O*-methylhydroxylamine hydrochloride (10 mg/mL MeOx in pyridine). Trimethylsilylation was performed by adding 40 µL of N-methyl-N-(trimethylsilyl) trifluoroacetamide (MSTFA) + 1% trimethylchlorosilane (TMCS), keeping the temperature constant at 70 °C for 60 min. The derivatized samples were cooled to room temperature prior to injection.

### 2.6. GC-MS Based Metabolomics Analysis of H. pinifolia SCC

Metabolomics analysis was performed to identify the polar metabolites induced in *H. pinifolia* SCC grown under artificial conditions. GC-MS was performed using an Agilent 7890A-5975C GC-MS system coupled with an HP-5MS capillary column (30 m × 0.25 mm) with fused silica coated with 0.25 mm CP-SIL 8 CB low beads. Then, 1 µL of derivatized sample was injected into the GC column using a TriPlus auto sampler with helium at a constant flow rate of 1 mL min^−1^. The injector was operated in a splitless mode and maintained isothermally at 230 °C. The temperature for the MS transfer line to quadruple was set at 280 °C, the electron impact ion source at 230 °C, and the MS QUAD at 100 °C. Compound elution from the plant extracts was set based on the following oven temperature program: injection temperature was set at 70 °C, followed by a 7 °C/min oven temperature gradient final to 325 °C, and holding for 3.6 min at the same temperature. The temperature in the GC-MS system was equilibrated for 1 min at 70 °C before injecting the next sample. Ions were generated by a 70 eV electron beam at an ionization current of 2.0 mA, and the spectra were recorded at 2.91 scans per second with a scanning range of 50–600 *m*/*z*.

### 2.7. HPLC Chromatographic Separation and Structural Elucidation of SCC Metabolites

Based on the standards, MeoH extracts of *H. pinifolia* SCC were checked for the desired fraction containing the compounds with higher concentration (*p*-anisic acid, *p*-coumaric acid, caffeic acid, vannilic acid, and rosmarinic acid) using an Agilent 1260 series preparative HPLC (Agilent Technologies Inc., Chemetrix) equipped with a binary pump, including a photodiode array detector. The separation was achieved by a reverse phase Acclaim TM 120 C18 column (Phenomenex; 4.6 × 250 mm, 5 mm particle size). The mobile phase contains 1% aqueous acetic acid solution (Solvent A) and acetonitrile (Solvent B). Flow rate was adjusted to 0.8 mL/min and the column was thermostatically controlled at 280 °C, whereas the injection volume was kept at 10 µL. A gradient elution was performed by varying the proportion of solvent B to solvent A, which was changed from 10 to 40% B in a linear gradient for the duration of 25 min, from 40 to 60% B of 38 min and from 60 to 90% B in 50 min. The mobile phase composition back to initial condition (solvent B: Solvent A: 10: 90) in 55 min and allowed to run for another 10 min, prior to the injection of another samples. The analysis per sample was carried out for 65 min. HPLC chromatograms were detected using photodiode array UV detector at three different wavelengths (260, 280 and 310) as per the absorption maxima of analysed compounds. Each compound was identified based on the retention time and by the spiking standards under the same conditions. Quantification of the sample was done by the measurement of the integrated peak area and the content was calculated using the calibration curve by plotting the peak area against concentration of the respective standard samples.

### 2.8. Alternative Screening Approach for Growth Inhibition of C. albicans in YNB Broth

For the primary screening, *H. pinifolia*-derived compounds (*p*-anisic acid, *p*-coumaric acid, caffeic acid, vanillic acid and rosmarinic acid) was used at a concentration of 1 mg/mL in order to determine the growth of *C. albicans* ATCC10231 in the YNBL medium supplemented with glucose or lactate as sole carbon source. FLC treatment was executed as positive control, and each plate included drug free wells as a negative control. The yeast was grown in each well at a final volume of 200 µL with a final cell density of 0.5–2.5 × 10^5^ cfu/ mL. The plates were then incubated at 35 °C for 24 h, and the OD was measured at 530 nm. Growth in each compound was calculated in percentage with respect to the drug free control. Similarly, a growth assessment in YNBG was also conducted based on the above procedure and all the experiments were done in triplicates. Compounds which inhibit the growth of *C. albicans* were further characterized for specific ICL inhibition as described below.

### 2.9. Isocitrate Lyase (ICL) Enzyme Assay and Inhibition Studies

*Candida albicans* was grown for 24 h in YNBL medium in order to induce the expression of ICL. The cells were harvested by centrifugation at 5000 rpm for 3 min. Cell-free extracts were prepared as per the methods of Cruz et al. (2011), with slight modifications. The harvested cells were washed with prechilled buffer (100 mM potassium phosphate buffer, pH 5; 2 mM MgCl_2_; 1 Mm DTT), followed by sonication for 3 min. It was then centrifuged and the cell pellet was collected. It was then resuspended with the same buffer, sonicated for 3 min with 0.7 mm glass beads. The cell lysate was centrifuged at 20,000 rpm for 20 min at 4 °C to obtain the cell free supernatant which was used in the enzyme assay.

An isocitrate lyase enzyme assay was carried out in 96-well plates. To 100 µL of cell free extract, 100 µL of the reaction mixture composed of 25 mM imidazole (pH 6.8), 5 mM MgCl_2_, 1 mM EDTA, 4 mM phenylhydrazine HCL, and 1 mM DL-isocitric acid (substrate). The addition of substrate results in the formation of glyoxylate phenyl hydrazone at 30 °C was absorbed at 324 nm using microplate absorbance reader (Bio-RAD model 680 microplate reader). Similarly, the reaction mixture was prepared without the substrate was serving as blank. In order to identify potent ICL inhibitors, compounds isolated from *H. pinifolia* (*p*-anisic acid, *p*-coumaric acid, caffeic acid, vannilic acid, and rosmarinic acid) were added to the reaction at a final concentration of 25 mg/mL, followed by incubation, and the OD was measured as described above. The percentage inhibition of ICL enzyme activity was calculated for each compound relative to the drug-free control.

### 2.10. Minimum Inhibitory Concentration of Identified Lead Compounds

The potential ICL inhibitors were subjected to MIC determination according to the procedure described by EUCAST Definitive Documents EDef 7.1. The cells at a density of 0.5–2.5 × 10^5^ cfu/mL were cultured in 96-well plates and separately treated with a gradient of concentration from 64 to 0.125 mg/L (for flucanazole) and 1000 to 1.95 mg/L (for ITC and potent inhibitors from *H. pinifolia*) in wells from column 1–10, respectively. The drug-free control was also calculated in order to determine the relative growth percentage. The cells were incubated, and the growth was measured allowed to incubate as previously described.

### 2.11. Drug Likeliness and ADMET Properties of RA

The drug likeliness was evaluated via Lipinski’s rule of five using Molinspiration software (http://www.molinspiration.com/cgi-bin/properties; accessed on 29 December 2021) to determine the molecular properties. The Lipinski’s rules (RO5) state that molecules exhibit good absorption and permeation when they have an octanol-water partition coefficient (Milog P) < 5, molecular weight < 500, number of hydrogen bond donars (n OHNH) ≤ 5, number of hydrogenbion acceptor (n ON) ≤ 10. The “rule of 5” provides a heuristic guide to determine whether the compound is orally bioavailable. The oral bioavailability and the membrane permeability properties have often been correlated to logP, molecular weight, and the number of hydrogen donors and acceptors in a molecule. The chemical structure of the potent inhibitors was submitted to the admetSAR server (http://www.admetexp.org/predict/ (accessed on 29 December 2021) for the in-silico prediction of absorption, distribution, metabolism, excretion, and toxicity (ADMET) properties [37].

### 2.12. Statistical Analysis

All experiments were carried out in triplicate, and the results are given as mean ± standard deviation. Treatment mean comparisons were performed using the least significance difference test. Data obtained from GC-MS were subjected to multivariate analysis using Metaboanalyst 3.0. To evaluate relationships among groups of variables, i.e., control and sample, normalised data were subjected to PLS-DA, and the output consisted of a score plot to show the contrast between different groups, whereas a correlation matrix was used to identify compound clusters.

## 3. Results

### 3.1. Callus Induction and Suspension-Cultured Cells of H. pinifolia

**Figure 1:**
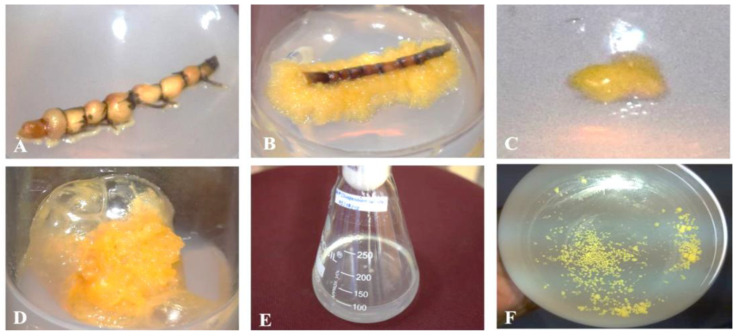
Callus induction and establishment of cellular suspension of *H. Pinifolia*. (**A**)—Explant; (**B**)—Callus induced from Explant; (**C**)—Callus subculture on 3rd day; (**D**)—Callus subculture on 21st day; (**E**)—Cell suspension culture as larger clump; (**F**)—Suspension cultured cells at granular stage.

Callus induction from inter-nodal rhizome segments of *H. pinifolia* was observed on day 12, with a fresh weight of 3.29 ± 0.02 mg, and reaching an average weight of 85.27 ± 1.36 mg. Calli induced from *H. pinifolia* were observed to be yellowish, friable, and irregular, as reported earlier by Jeyapragash et al. [34], and remained the same even after sub-culturing. However, in the present study, calli were induced on the 8th day when the medium was supplemented with the same concentration of 1.5 mg/L 2,4-D, 1 mg/L BAP, and 4% sucrose. Suspension-cultured cells of *H. pinifolia* was established by transferring friable calli to MS liquid medium with continuous shaking in order to disintegrate the larger clumps to the granular stage. The callus and cellular suspension of *H. pinifolia* are shown in Figure 1. To achieve reproducible growth cycles of cell suspension cultures, measuring growth parameters is important; thus, the growth characteristics of *H. pinifolia* suspension-cultured cells upon MeJA elicitation was studied.

### 3.2. Influence of MeJA Concentration on Rosmarinic Acid Biosynthesis

An increase in RA accumulation was observed in all applied elicitor concentrations, but the maximum accumulation was found in the *H. pinifolia* suspension treated with 20 µM MeJA on the 3rd day after inoculation. The fresh and dry weights of the cells obtained were 43.5 ± 2.7 g L^−1^ and 3.45 ± 0.31 g L^−1^ at day 3 after MeJA inoculation, and reached a maximum of 257.33 ± 5.19 g L^−1^ and 39.53 ± 2.08 g L^−1^ at day 21, with decreased concentrations recorded on subsequent days. The accumulation of RA was constantly increased until 21 days after elicitation, where the maximum concentration of RA (117 mg/g dry weight of cells) was achieved upon MeJA (20 µM) treatment.

### 3.3. GC-MS Based Metabolomics Analysis Following MeJA Elicitation

Metabolites synthesized from *H. pinifolia* cells following MeJA induction (20 µM) were analyzed by gas chromatography (GC)-MS, and multivariate analysis was performed. Low molecular weight metabolites from *H. pinifolia* cells were identified and the peak locations were determined using ChromeTOF software. Metabolite peak identification was performed using standards and the in-house library, whereas amino acids and sugars were identified by mass chromatograms with commercially available standards. In total, 47 metabolites which were found to be significantly different between the control and MeJA-treated cells were profiled from *H. pinifolia*, including 30 organic acids, six amino acids, six flavonoids, two sugars, two plant growth regulators, and one vitamin.

**Figure 2:**
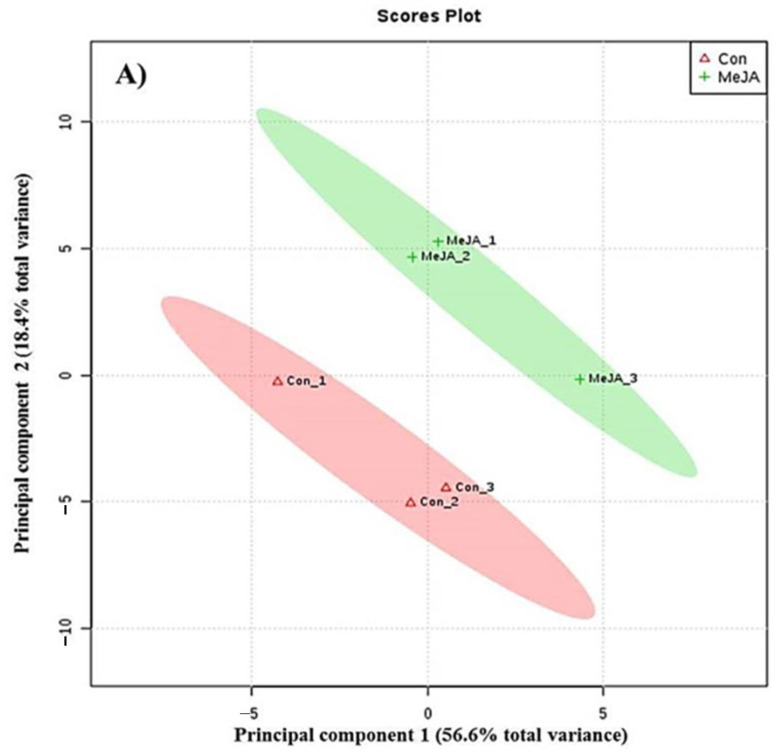

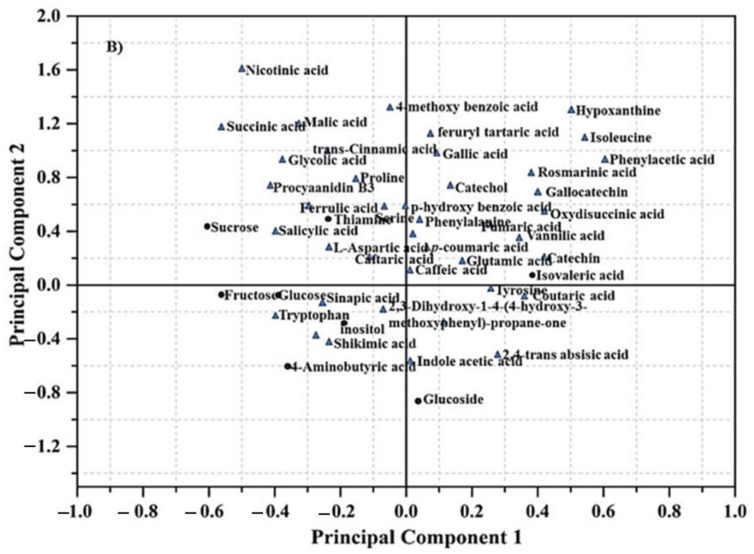
PLS-DA results of score (**A**) and loading plots (**B**) of principal component 1 and 2 for polar metabolites data obtained from MeJA-treated cells and control cells of *H. Pinifolia*.

PLS-DA showed a clear metabolite grouping distribution of control and MeJA-treated cells, as shown on the score plot (Figure 2A). MeJA-treated cells were strongly influenced by the observations in the upper right quadrant compared with control cells. The PLS-DA loading plot exhibited the magnitude and direction of correlations of the original variables with principal components. The results of PLS-DA provide a clear difference between the control and MeJA-treated cells, with an obvious separation pattern of metabolic data. The first principal component resolved the metabolites of control and MeJA-treated cells, and accounted for 56.6% (PC1) of the variation, whereas the second component accounted for approximately 18.4% (PC2) of the variation. The goodness of prediction values for control (R^2^X > 0.629; R^2^Y > 0.810 and Q^2^ > 0.632) and MeJA-treated cells (R^2^X > 0.698; R^2^Y > 0.891 and Q^2^ > 0.820) were obtained, and indicated that the models could predictably reveal the metabolomic variations between the different groups of control and MeJA-treated cells (Figure 2A). In total, 47 metabolites were profiled, among which, 30 are organic acids, six are amino acids, six are flavonoids, two are sugars, two are plant growth regulators, and one is a vitamin, which were found to be significantly different between the control and MeJA-treated cells, respectively. As compared to the control, MeJA-treated cells induced a higher number of metabolites, since MeJA acting as an elicitor induced the maximum number of organic compounds at a higher concentration (Figure 2B). The present study indicates that the concentration of phenolic acids and indole-acetic acid in MeJA-treated cells increased compared with control groups, whereas the concentration of sugars and aromatic amino acids decreased. All of the phenolic acids, such as RA, gallic acid, *p*-coumaric acid, and glutamic acid, as well as indole-acetic acid, were clustered in the right of the loading plots, which was strongly influenced by the observations, whereas aromatic amino acids, and sugars such as glucose and sucrose, were clustered in the left of the loading plots. The results of the present study indicate that the levels of organic acids, such as *p*-anisic acid, *p*-coumaric acid, caffeic acid, vanillic acid, and rosmarinic acid, in MeJA-treated cells were increased compared with control groups. Further, the purification of organic acids by HPLC and structurally elucidation was performed by NMR for biomedical applications.

### 3.4. HPLC Chromatographic Separation and Structural Elucidation of SCC Metabolites

**Figure 3:**
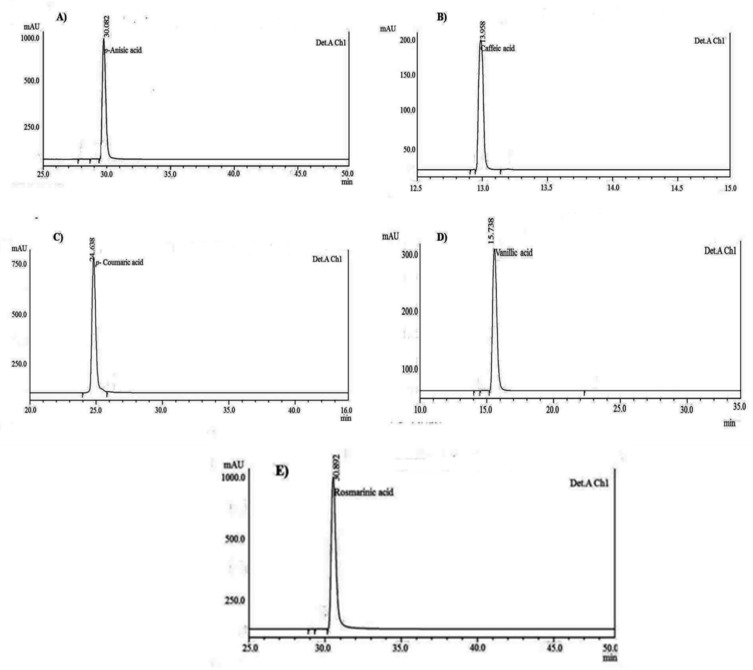
HPLC chromatogram for purity check of individual compounds isolated from the extracts of *H. pinifolia.* (**A**). *p*-Anisic acid; (**B**). Caffeic acid; (**C**). *p*-Coumaric acid; (**D**). Vanillic acid; (**E**). Rosmarinic acid.

It was observed that all five compounds showed the UV absorption range, and *p*-anisic acid was detected at a wavelength of 284 nm, *p*-coumaric acid at 260 nm, caffeic acid at 280 nm, vanillic acid at 299 nm, and rosmarinic acid at 330 and 260 nm, respectively. In order to detect the presence of compound impurity from TLC, it was further subjected to HPLC, and the purity was visualized from the analytical peaks. The chromatogram of each compound showed the absence of shoulder, valley, or excessive tailing, which confirmed that the compounds are pure. The absence of these features on the chromatogram sometimes may not act as a foolproof assurance of peak purity because the resolution of the chromatogram is low, and hence, photodiode array was used to determine the peak purity of each compound isolated from *H. pinifolia.* The purity of each compound obtained was in the range of 97.29% to 99.17%. The chromatogram for pure phenolic acids and flavonoids is shown in Figure 3A–E. The concentration of eight compounds was found to be the maximum in SCC of *H. pinifolia* rather than wild samples. In the wild sample, *p*-coumaric acid was found to be accumulated at a concentration of 55.72 ± 8.2 µg/g, followed by rosmarinic acid (39.28 ± 2.5 µg/g), *p*-anisic acid (21.53 ± 3.7 µg/g), and caffeic acid (9.74 ± 1.08 µg/g), and minute concentrations of other compounds were obtained, whereas in the SCC sample, rosmarinic acid accumulated at a higher concentration (39.27 ± 4.73 mg/g), followed by caffeic acid (12.79 ± 2.35 mg/gm), *p*-anisic acid (11.37 ± 2.19 mg/g), *p*-coumaric acid (10.98 ± 1.07 mg/g), and vanillic acid (8.32 ± 1.37 mg/g), respectively. Hence, the compounds purified from *H. pinifolia* were subjected to ^1^H and ^13^C NMR, and the structures of the compounds were confirmed based on the earlier reports.

### 3.5. Alternative Screening Approach for C. albicans Growth in YNB Broth

In this study, the growth of *C. albicans* in YNBL medium was determined, and the cut-off value was set at 40% growth reduction with respect to the drug-free control. Itaconic acid and fluconazole were used as positive controls. Among the five compounds, rosmarinic acid showed the minimum growth percentage of 1.99%, as it inhibited the growth of *C. albicans*, followed by *p*-anisic acid (34.4%), caffeic acid (48.2%), *p*-coumaric acid (76.3%), and vanillic acid (85.4%).

**Figure 4:**
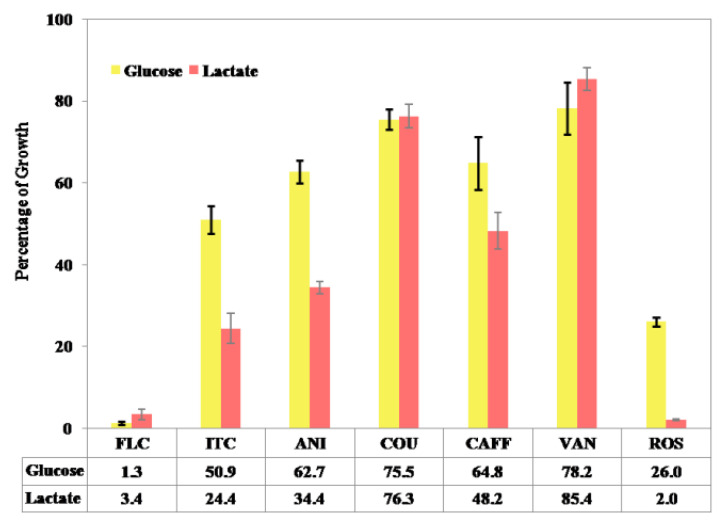
Alternative screening approach for *C. albicans* growth in YNB broth supplemented with glucose or lactate as sole carbon source. Error bar in the chart represents standard deviation. (FLC—Fluconazole; ITC—Itaconic acid; ANI—*p*-Anisic acid; COU—*p*-Coumaric acid; CAFF—Caffeic acid; VAN—Vanillic acid; ROS—Rosmarinic acid).

Furthermore, growth assessment in YNBG medium helped to get a narrow list of potential ICL inhibitors (rosmarinic acid, *p*-anisic acid, and caffeic acid) that showed a lactate-specific pattern of growth reduction similar to itaconic acid. In contrast, vanillic acid showed a glucose-specific pattern of *C. albicans* growth (78.2%) similar to fluconazole, whereas *p*-coumaric acid exhibited their growth in both of the media (YNBL—76.3%; YNBG—75.5%), which indicates non-specific inhibition or different targets (Figure 4).

### 3.6. ICL 1 Enzyme Assay Confirmed Potential Inhibitors

**Figure 5:**
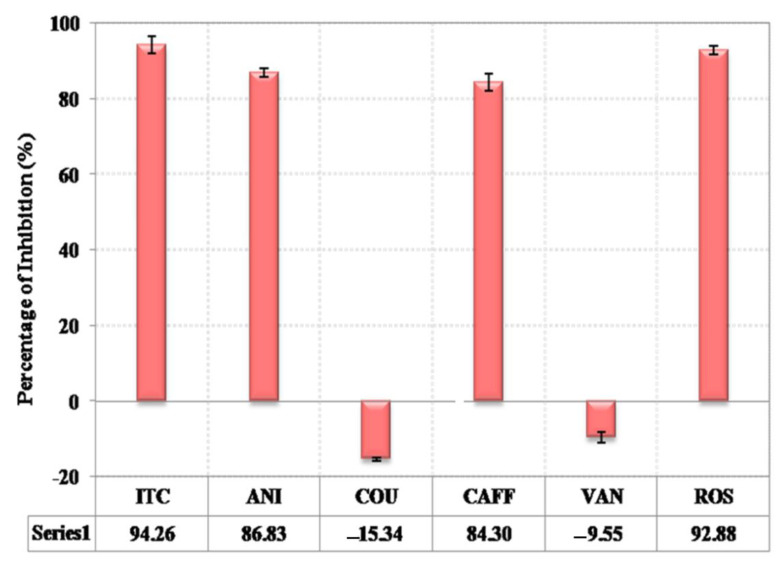
Percentage Inhibition of *H. pinifolia*-derived compounds in the ICL 1 enzyme inhibition assay.

The induction of ICL 1 in *C. albicans* was performed to formulate cell-free extract for use in the ICL assay and inhibition studies. Spectrophotometric determination confirmed the expression of ICL 1 by forming glyoxylatephenylydrazone, which showed an absorbance value of 0.71 µg/100 µL. Further studies on ICL 1 inhibition using *H. pinifolia*-derived compounds were carried out, and the cut-off value for enzyme inhibition was set at 40%, and itaconic acid was used as positive control.The known ICL inhibitor and some compounds showed inhibitory percentages higher than 40%. Romarinic acid showed the maximum inhibition of ICL 1 enzyme with 93.1%, followed by *p*-anisic (86.2%) acid and caffeic acid (85.1%). Other compounds did not show any inhibition on ICL 1 enzyme in *C. albicans* (Figure 5). Rosmarinic acid, *p*-anisic acid, and caffeic acid were identified as potential ICL inhibitors in the alternative screening experiments that showed ICL enzyme inhibition similar to the positive control, with an inhibitory percentage higher than 40%.

### 3.7. Determination of Minimum Inhibitory Concentration

The minimum inhibitory concentration of tested compounds was obtained in YNBL broth using the broth microdilution method. Fluconazole and itaconic acid were used as positive control, and the MIC was found at a concentration of 16 mg/L and 250 mg/L. The MIC determination for potential inhibitors was found to be 31.25 mg/L for rosmarinic acid, 500 mg/L for *p*-anisic acid, and 1000 mg/L for caffeic acid (Table 1).

### 3.8. Drug Likeliness and ADMET Properties of RA

The molecular properties of known ICL 1 inhibitors and potential inhibitors isolated from *H. pinifolia* are represented in Table 2. The canonical smiles of fluconazole, itaconic acid, *p*-anisic acid, caffeic acid, and rosmarinic acid were submitted to the Molinspiration server, and the drug-likeliness was determined. The compounds tested for drug-likeliness were roughly submissive with Lipinski’s rule of five. The total polar surface area of all the tested compounds was found in the range of 46.53 to 144.52, and well below the 160 A limit. The octanol:water partition coefficient was found to be less than 5 for all the tested compounds, whereas the number of hydrogen bond donors (OH and NH groups) and acceptors (O and N) was found to be within the Lipinski’s rule limit, i.e., less than 5 and 10, respectively. However, all the compounds showed no violations with respect to molecular weight, lipophilicity (mi LogP), number of hydrogen bond donors, and the number of hydrogen bond acceptors. The mi LogP value was found to be applicable to Lipinski’s rule, and it has the properties to use it as a drug.

ADMET properties have been conducted to determine the bioavailability and toxicity of the compounds in order to use them as drugs (Table 3). The blood–brain barrier levels of all compounds reflect high to medium penetration level, except caffeic acid, whereas the human intestinal absorption of all compounds showed good absorption levels, respectively. Caco-2 permeability of tested compounds showed positive results, except rosmarinic acid and known ICL inhibitor itaconic acid. Rosmarinic acid was found to act as a substrate for P-glycoprotein, and the cytochrome *p* inhibitory promiscuity was found to be low for all the tested compounds. The compounds submitted in the admetSAR software showed weak inhibition of human ether-go-go-related genes (HERG), and have not showed any toxicity and carcinogenicity during their metabolism. Based on the results obtained from ADMET properties, the bioactivity scores of all compounds were determined.

The bioactivity scores of screened compounds were predicted and compared with standard drugs (drug targets, such as GPCR ligand, ion channel modulator, kinase inhibitor, protease inhibitor, and nuclear receptor ligand and enzyme inhibitory activity, are summarized in Table 4). The results demonstrated that the tested compounds were found to be biologically active, and produced physiological actions by interrelating with GPCR ligands, nuclear receptor ligands, and hindering other enzyme activity. Rosmarinic acid (0.17) exhibited the highest bioactivity score for the drug target GPCR ligand with respect to the positive controlfluconazole (0.04). Ion channel inhibitor was found in between −0.08 to 0.97, of which, positive control fluconazole and itaconic acid showed higher interaction with this target than test compounds, whereas similar results were obtained for kinase inhibition. In this study, rosmarinic acid showed a bioactivity score of −0.18, which was found to be closer to the positive control fluconazole (−0.09), whereas other compounds were inactive towards this drug target. The compounds bioactivity score with the nuclear receptor ligand, protease inhibitor, and enzyme inhibitor ranged from −0.10 to 0.57, −0.09 to 0.15, and −0.09 to 0.24 exhibited high bioactive nature of molecule. Of the compounds isolated from *H. pinifolia* and the positive control studied, rosmarinic acid exhibited the most promising result, with a bioactivity score of 0.57, 0.15, and 0.24 for nuclear receptor ligand, protease inhibitor, and enzyme inhibitor.

## 4. Discussion

Metabolomics is a promising holistic approach primarily driven by recent advances in mass spectrometry (MS) technology, and also by the goals of functional genomics. Nevertheless, achieving a broad overview of the metabolic composition of an organism is very complex, and a metabolomics approach has been established with a multifaceted, full integrated strategy for optimal sample extraction, metabolite separation detection, automated data gathering, and, ultimately, with quantification. Though numerous mass spectrometry-based metabolomics have been reported so far, the GC-MS-based approach was found to be appropriate to all kinds of biological systems, and allows the unbiased identification of novel metabolites and biomarkers embedded in correlative metabolite–protein networks. Taken together, metabolomics was found to have significant applications in the diagnostic characterization of metabolic features under different genetic and environmental conditions, and also helps to understand the complex changes occuring under certain circumstances. Seagrasses are specialized with unique ecological, physiological, and morphological adaptations to a completely submerged marine existence, by sharing the most features of primary and secondary metabolism. Seagrass harbors a wide variety of secondary metabolites that attribute to the bioactive potential, and it has gained limelight as a potential antioxidant, antibacterial, antifungal, antiviral, antidiabetic and vasoprotective agent [35]. Though the literature documented the chemistry of seagrasses, some interesting aspects on their metabolomics approach and MeJA-induced rosmarinic acid biosyhthesis have recently addressed [36,37]. However, the stress responsive metabolomics of in-vitro cultured cells of seagrasses have not been sufficiently addressed. Furthermore, no reports are available on the human ortholog from seagrass metabolites as a promising target to treat *C. albicans* infection. Since the lead compounds may have highly complex structures with many chiral centers, it has not been attempted for chemical synthesis. So as to conserve the seagrass community and prevent the loss in compound activity during chemical synthesis, the lead compounds or inhibitors from seagrasses need to be biosynthesized by means of plant tissue culture technology.

The callus induction and suspension-cultured cells of *H. pinifolia* were established and were observed to be yellowish, friable, and irregular, as reported earlier by Jeyapragash et al. [34]. Calli were induced on the 8th day when the medium was supplemented with 1.5 mg/L 2,4-D, 1 mg/L BAP, and 4% sucrose, which was in contrast to Subhashini et al. [32] and Jeyapragash et al. [34]. The first report on the induction of calli from seagrass *H. wrightii* used Anderson’s stage I medium supplemented with IBA, NAA, and kinetin with sucrose, coconut milk, arginine, and glutamine [37]. It was followed by Subhashini et al. [32], who showed that *H. pinifolia* exhibited friable calli when the MS medium was supplemented with 2 mg/L 2, 4-D and 1 mg/L BAP. In the present study, calli were induced within 12 days when the medium was supplemented with 1.5 mg/L 2,4-D, 1 mg/L BAP, and 4% sucrose. It has been reported that the type and concentration of the plant hormone alter the induction and growth of cells in vitro [38,39]. The findings of Wang et al. [40] support the establishment of suspension cultures to grow cells continuously and stimulate the formation of pro-embryonic and pro-organogenic calli. Cellular suspension of *H. pinifolia* was obtained by transferring the friable calli to MS liquid medium. The suspended calli disintegrated to form larger clumps by continuous shaking, and attained a yellowish granular stage. The callus and cellular suspension of *H. pinifolia* are shown in Figure 1. To achieve reproducible growth cycles of cell suspension cultures, measuring growth parameters is important; thus, the growth characteristics of *H*. *pinifolia* suspension-cultured cells upon MeJA elicitation was studied.

Several studies on cell cultures from various plant species have shown that elicitation enhances the specialized metabolite production. Nevertheless, the parameters, such as elicitor specificity, its concentration and time of its exposure, as well as the growth stage of cultured cells and the culture condition, influence the elicitation processes [41]. A recent study also found that the concentration of MeJA considerably elevated RA accumulation in *Coleus blumei* [42] and *Lithospermum erythrorhizon* [43]. It has also been reported that the accumulation of RA was induced by the yeast elicitor when it was added to *L. erythrorhizon* suspension cells [43] and *Orthosipho naristatus* culture [44]. Hippolyte et al. [21] reported that 5% sucrose resulted in maximum RA accumulation in suspension cultured cells of *S. officinalis.* By contrast, the *Ocimum sanctum* cell suspension culture exhibited lower biomass and RA accumulation at higher sucrose concentration, which might be due to high osmolarity that could lead to metabolic changes in hypertonic conditions. Another study showed that the suspension culture of *O. sanctum* exposed to MeJA (100 mM) enhanced the cell growth and RA accumulation, which is similar to the present study [25], and the present study enhanced the RA production at a higher concentration.

Omics-approach-based metabolomics analysis of *H. Pinifolia* SCC revealed a total number of 47 metabolites, which is significantly different between the control and MeJA treated cells, respectively. The present study indicates that the concentration of phenolic acids and indole-acetic acid in MeJA-treated cells increased compared with control groups, whereas the concentrations of sugars and aromatic amino acids were decreased. The present study is consistent with the earlier report of Kim et al. [45], where MeJA treated with *A. rugosa* increased the levels of phenolic acids and aromatic amino acids, whereas the concentration of sugars and threonic acid decreased following treatment with MeJA. Liang et al. [46] also reported that the concentrations of hydroxylcinnamates and glucosinolate were elevated, and glucose, sucrose, and amino acids decreased when *Brassica rapa* leaves were treated with MeJA. It has also been reported that the primary metabolic pool was observed in fewer sucrose and MeJA- and yeast-treated samples than those in controls. This might be a consequence of the fundamental metabolic repartitioning of carbon sources rather than specific induction elicited by the stimuli [47]. In addition to the specialized products synthesized by *H. pinifolia* cells upon MeJA induction, several primary metabolites also accumulated, such as succinic acid, glucoside, tryptophan, and L-ascorbic acid, to greater levels than the control cells. The accumulation patterns for these primary metabolites cannot be explained via common catabolic phenomena or by their ecological functions. The observations in the present study suggest that MeJA could induce cells to synthesize a greater number of organic acids with a greater accumulation of RA, as well as cause rapid changes in photosynthate pools, thus helping to increase partitioning of new carbon into amino acids. It has also been reported that MeJA can impart selective control over the shikimate pathway, which gives rise to differential partitioning of new carbon into its metabolites [45].

HPLC determination of phenolic acid and flavonoids was determined based on the peak area of standards. Though more than a hundred kinds of ultraviolet absorptive organic compounds would be contained in a wild and SCC extracts of *H. pinifolia,* the analysis of phenolic acids and flavonoids such as *p*-anisic acid, caffeic acid, *p*-coumaric acid, vanillic acid, rosmarinic acid, protocatacheuic acid, 4-hydroxybenzoic acid, and naringenin, were detected based on the suitable wavelength of the standards. In the present study, three rules have been followed during the HPLC analysis, which includes the detection of an ultraviolet absorbance band of each phenolic acid, which should be set at the maximum absorbance wavelength; the wavelength which was absorbed by most compounds was preferred; and finally, the detection wavelength for different compounds were merged as much as possible in order to simplify the data processing. In the present study, all standard compounds showed maximum absorpotion at 260 nm, which was further used for the detection of analytes in the *H. pinifolia* sample. Wang et al. [48] described a simple and reproducible HPLC method, where the recovery rate of rosmarinic acid and caffeic acid was found to be 27.1 mg/g and 1.8 mg/g, which are comparably lower than the present study, as the SCC sample of *H. pinifolia* accumulated rosmarinic acid at a concentration of 39.27 ± 4.73 mg/g, and caffeic acid as 12.79 ± 2.35 mg/gm. Li et al. [49] used four different wavelengths for the detection of analysts from herbal samples. The conditions selected in the study of the herbal sample were: 254 nm for anisic and vanillic acid; 275 nm for gallic acid, trans-cinnamic acid, and syringic acid; 305 nm for salysilic acid; and 320 nm for chlorogenic acid, gentisic acid, caffeic acid, sinapic acid, *p*-coumaric acid, ferulic acid, and rosmarinic acid. Further, Seal [50] separated the investigated compounds from the leaf extracts of *O. linearis* and *S. arvensis* at 272 nm based on the HPLC chromatogram of all standard mixtures. As compared to the earlier reports, the rate of recovery of the standards and the investigated analytes from *H. pinifolia* were found to be significant and worth mentioning.

Isocitrate lyase (ICL) is one of the key enzymes in the TCA cycle, which helps *C. albicans* to grow and survive in a niche devoid of glucose, and cause candidiasis [51,52,53]. It was recently reported that ICL acts as a virulence of numerous pathogen, and it has been discovered as a potential target for drug discovery [54,55,56]. Moreover, there is no known human ortholog of this enzyme and, hence, it can be considered as a promising antifungal target to treat candidiasis with minimal hostile side effects and toxicity. In the present study, the five potential compounds (*p*-Anisic acid, *p*-coumaric acid, caffeic acid, vanillic acid, and rosmarinic acid) obtained from primary screening were screened secondarily using an alternative approach in order to identify the potential ICL inhibitors.

Conventional methods of antifungal screening mostly utilize a medium enhanced with glucose as carbon source [57,58], and hence, this method may not be relevant for the host niches, which might cause the loss of potential of certain target specific compounds. Therefore, the present study used minimal defined medium supplemented with lactate as a substitute of glucose in order to simulate the environment where *C. albicans* grows in host niches. The alternative screening approach resulted with three potential ICL inhibitors, and rosmarinic acid showed the minimum growth percentage of 1.99%, as it inhibited the growth of *C. albicans*, followed by *p*-anisic acid (34.4%) and caffeic acid (48.2%). All three compounds showed a lactate-specific pattern of growth reduction. *p*-coumric acid and vanillic acid were found to be non-specific, and not associated with the TCA cycle to target the cell activity. It was found that vanillic acid showed a glucose-specific growth pattern, which suggests that it might have targeted the metabolic pathway associated with glucose assimilation. Similarly, rutin, a plant-derived metabolite, was reported to exhibit a glucose-specific growth pattern while grown in YNBG and YNBL medium [53]. Hence, this secondary screening helped the present study to avoid the interaction of any non-specific detergent that reduced the growth of *C. albicans* by cell lysis or toxicity rather than a defined mechanism of action.

In order to confirm the growth inhibitory property of five potential compounds, it was then screened for the inhibition of ICL enzyme activity. The known ICL inhibitor and the compounds showed an inhibitory percentage higher than 40%. Rosmarinic acid showed the maximum inhibition of ICL 1 enzyme with 93.1%, followed by *p*-anisic (86.2%) acid and caffeic acid (85.1%). The results from this study are not as certain, as they might inhibit ICL activity in combination with other cellular components from the microbial crude extract, because ICL activity regulation in the yeast will get activated at the post-translational level [59]. Furthermore, the purification of ICL enzyme was not executed in order to prevent the inactivation or degradation of the enzymes. The three potential ICL inhibitors were further subjected to *C. albicans* MIC determination using YNBL medium. The MICs were found to be lower for positive control fluconazole (16 mg L^−1^), rosmarinic acid (31.25 mg L^−1^), itaconic acid (250 mg L^−1^), *p*-anisic acid (500 mg L^−1^), and caffeic acid (1000 mg L^−1^). The high MICs value obtained for vannilic acid and *p*-coumaric acid suggest that over-dosage of those compounds might have resulted with growth inhibition due to general toxicity. In contrast to this study, Cheah et al. [53] reported that caffeic acid and rosmarinic acid showed high MICs values for *C. albicans* treated with 1000 mg/L.

One of the requirements for a drug discovery is to identify the potential biological activity at the target of interest. Screening enormous number of compounds has increased over the past 10 to 15 years, and the corresponding increase in the successful launching of drugs has not yet been ensued. The lack of success in drug discovery is due to the failure rate at the clinical trial stage transition from Phase II to Phase III due to toxicity, adverse side effects on the recipients, and poor ADMET properties [60]. Hence, more attention has to be paid on ADMET properties, especially during the drug safety assessments, and it is significant for reducing the failure rate at the clinical stage. Though the experimental assessment of ADMET properties is expensive, labor intensive, and did not meet the demands of high throughput drugs, in-silico techniques can easily predict the ADMET properties depending on the structure activity relationship of a test compound. In this study, the canonical smiles of potential inhibitors were submitted in Molinspiration admetSAR servers and analyzed the drug-likeliness of a test compound based on Lipinski’s rule of five [61]. The present study showed that none of the potential inhibitors violated any of the rules. Though Lipinski’s rule-of-five is useful in early drug assessment, the properties of a drug administered into the recipient are significant to know at later stages of valuation process [53].

The analysis of ADMET properties performed on the admetSAR revealed that the potential inhibitors showed low CYP inhibitory promiscuity, as it loses the inhibition of most cytochrome p450isoforms, including CYP450 1A2, 2C9, 2C19, and 3A4. The cytochrome P450 superfamily plays a vital role in drug metabolism and prevents the accumulation of drugs in the liver, and the most significant isoforms are CYP1A2, CYP2A6, CYP2C19, CYP2D6, CYP2E1, and CYP3A4. The analysis also showed that rosmarinic acid acts as a substrate for P-glycoprotein, which helps to efflux a drug and other compounds in order to metabolize further for clearance [62]. Drugs in the medications would be transported out of the cells at a greater rate when P-glycoprotein is induced [62], which leads to therapeutic failure, owing to the lower concentration of the drug than the expected, and so, the dosage control, as well the information of co-administered drugs, should be considered to reduce the therapeutic failure. ADMET properties of the potential ICL inhibitors exhibited no significant potential for toxicity to humans. It has been reported that caffeic acid has been used as an active antioxidant and also an inhibitor of carcinogenesis [63]. However, some studies on caffeic acid toxicity reported to have mixed results that it inhibits carcinogenesis, whereas other experiments resulted with the provoking of a carcinogenic effect in laboratory mice [64]. Rosmarinic acid is a caffeic acid ester that acts as a defense compound [65], and earlier reports demonstrated that it has a number of biological activities [66]. However, toxicity studies on rosmarinic acid and *p*-anisic acid are scare in the literature.

Of the three potential ICL inhibitors studied, the lead compounds with high target selectivity, low toxicity, and better bioavailability, the present study effectively screened the molecular properties and bioactivity scores of rosmarinic acid, caffeic acid, and *p*-anisic acid. If the bioactivity score of a molecule is greater than 0.00, it is likely to possess considerable biological activity, whereas values of −0.50 to 0.00 are expected to be moderately active, and if the score is less than −0.50, it is inactive [67]. In the present study, rosmarinic acid (0.17) was found to exhibit the highest bioactivity score for drug target GPCR ligands, followed by the positive control fluconazole (0.04). Similar results were obtained for kinase inhibition, and rosmarinic acid showed a bioactivity score of −0.18, which was found to be closer to the positive control fluconazole (−0.09), whereas other compounds were inactive towards this drug target. Of the test compound and positive control studied, rosmarinic acid exhibited the most promising result, with a bioactivity score of 0.57, 0.15, and 0.24 for the nuclear receptor ligand, protease inhibitor, and enzyme inhibitor.

## 5. Conclusions

The present study reports the metabolomic features of different solvent extracts from wild and SCC of *H. pinifolia*. Moreover, five metabolites, such as *p*-anisic acid, *p*-coumaric acid, caffeic acid, vanillic acid, and rosmarinic acid, were found to accumulate at a higher concentration in the SCC of *H. pinifolia*. These observations show that 20 µM MeJA enhanced RA accumulation and showed a maximum level of RA biosynthetic gene expression as compared with the control. Moreover, the study attempted to identify the potential antifungal drugs that are effective against *C. albicans* by targeting ICL 1 enzyme of the glyoxylate cycle, using an alternative screening approach in a minimal defined medium with lactate as carbon source. Our resultsshow that rosmarinic acid derived from *H. pinifolia* SCC are identified as potential ICL inhibitors of *C. albicans*. Furthermore, studies need to be conducted to explore the compound in further detail in order to comprehend its activity against *C. albicans*, and its potential to cause adverse side effects in recipients, which will pave the way to consider rosmarinic acid as a lead viable drug for *C. albicans* infections. The present study shows that the compounds derived from *H. pinifolia* SCC upon elicitation can be identified as lead candidates for *C. albicans* infection by comparing the existing reference compounds, and that new pathways can be exclusively targeted using alternative target-based screening approaches.

## Figures and Tables

**Table 1:** MIC determination of potential ICL 1 inhibitor in *C. albicans*.

Potential ICL Inhibitors/Test Compounds	Minimum Inhibitory Concentration (mg/L)
Fluconazole	16
Itaconic acid	250
*p*-Anisic acid	500
Caffeic acid	1000
Rosmarinic acid	31.25

**Table 2:** Lipinski’s rule-of-five drug-likeliness properties of potential ICL inhibitors.

Compounds	Fluconazole	Itaconic Acid	*p*-Anisic Acid	Caffeic Acid	Rosmarinic Acid
**mi LogP**	−0.12	−0.343	1.91	0.941	1.63
**TPSA**	81.664	74.598	46.53	77.755	144.52
**n atoms**	22	9	11	13	26
**MW**	306.276	130.099	152.15	180.16	360.32
**n ON**	7	4	3	4	8
**n OHNH**	1	2	1	3	5
**n violations**	0	0	0	0	0
**n rotb**	5	3	2	2	7
**MV**	248.97	111.171	136.59	154.50	303.54

Abbreviations: mi LogP—Hydrophobicity measurement: octanol:water partition coefficient; TPSA—Topological polar surface area; MW—Molecular weight; n ON—Hydrogen bond acceptor; n OHNH—number of hydrogen bond donor; n violations—number of Lipinski’s rule-of-five violations; n rotb—number of rotatable bonds; MV—Molecular volume.

**Table 3:** ADMET properties of potential ICL 1 inhibitors in *C. albicans*.

S. No	Admet	Fluconazole	Itaconic Acid	*p*-Anisic Acid	Caffeic Acid	Rosmarinic Acid
**1.**	**Absorption**					
	BBB	+	+	+	-	+
	HIA	+	+	+	+	+
	Caco-2 permeability	+	-	+	+	-
	Aqueous solubility	−1.8626	−0.7363	−1.8579	−1.6939	−3.2050
	P-glycoprotein					
	Substrate	-	-	-	-	+
	Inhibitor	-	-	-	-	-
	ROCT	-	-	-	-	-
**2.**	**Distribution**					
	Subcellular localization	Mitochondria	Mitochondria	Mitochondria	Mitochondria	Mitochondria
**3.**	**Metabolism**					
	CYP450 Substrate					
	CYP450 2C9	-	-	-	-	-
	CYP450 2D6	-	-	-	-	-
	CYP450 3A4	-	-	-	-	-
	CYP450 Inhibitor					
	CYP450 1A2	-	-	-	-	-
	CYP450 2C9	+				
	YP450 2D6	-	-	-	-	-
	CYP450 2C19	-	-	-	-	-
	CYP450 3A4	-	-	-	-	-
	CYP Inhibitory Promiscuity	Low	Low	Low	Low	Low
**4.**	**Excretion and Toxicity**					
	HERG Inhibition					
	HERG 1	Weak	Weak	Weak	Weak	Weak
	HERG 2	-	-	-	-	-
	AMES Toxicity	-	-	-	-	-
	Carcinogens	-	-	-	-	-
	Rat Acute Toxicity (LD50, mol/kg)	2.4136	2.4525	1.8917	1.4041	2.6983
	Fish Toxicity (pLC50, mg/L)	1.4529; high	0.8180; high	1.9309; high	0.7921; high	−0.1231; high

Abbreviations: BBB—Blood–brain barrier; HIA—Human intestinal absorption; ROCT—Renal organic cation transporter; CYP450—Cytochrome *p* 450; HERG—Human ether a-go-go-related genes.

**Table 4:** Bioactivity score of test compounds as potential ICL 1 inhibitors.

Drug Target	Fluconazole	Itaconic Acid	*p*-Anisic Acid	Caffeic Acid	Rosmarinic Acid
**GPCR ligand**	0.04	−1.78	−1.02	−0.48	0.17
**Ion channel Modulator**	0.01	0.97	−0.55	−0.23	−0.08
**Kinase inhibitor**	−0.09	−2.82	−1.22	−0.81	−0.18
**Nuclear receptor ligand**	−0.23	−1.54	−0.79	−0.10	0.57
**Protease inhibitor**	−0.09	−2.08	−1.16	−0.79	0.15
**Enzyme inhibitor**	0.03	−1.23	−0.52	−0.09	0.24

## Data Availability

Not applicable.
